# The influence of color vision deficiency on vessel visibility during colorectal endoscopic submucosal dissection and the potential advantage of red dichromatic imaging to achieve color vision barrier‐free

**DOI:** 10.1002/deo2.410

**Published:** 2024-07-19

**Authors:** Akiko Ohno, Naohiko Miyamoto, Ryosuke Kaji, Takahiro Shirakawa, Moegi Watanabe, Ryutaro Sumi, Yoko Jinbo, Mitsunori Kusuhara, Jun Miyoshi, Tadakazu Hisamatsu

**Affiliations:** ^1^ Department of Gastroenterology and Hepatology Kyorin University School of Medicine Tokyo Japan; ^2^ Endoscopy Division National Cancer Center Hospital Tokyo Japan

**Keywords:** colorectal neoplasms, color vision deficiency, diversity, endoscopic submucosal dissection, universal design

## Abstract

**Objectives:**

Although color information is important in gastrointestinal endoscopy, there are limited studies on how endoscopic images are viewed by people with color vision deficiency. We aimed to investigate the differences in the visibility of blood vessels during endoscopic submucosal dissection (ESD) among people with different color vision characteristics and to examine the effect of red dichromatic imaging (RDI) on blood vessel visibility.

**Methods:**

Seventy‐seven pairs of endoscopic images of white light imaging (WLI) and RDI of the same site were obtained during colorectal ESD. The original images were set as type C (WLI‐C and RDI‐C), a common color vision. These images were computationally converted to simulate images perceived by people with color vision deficiency protanope (Type P) or deutanope (Type D) and denoted as WLI‐P and RDI‐P or WLI‐D and RDI‐D. Blood vessels and background submucosa that needed to be identified during ESD were selected in each image, and the color differences between these two objects were measured using the color difference (Δ*E*_00_) to assess the visibility of blood vessels.

**Results:**

Δ*E*_00_ between a blood vessel and the submucosa was greater under RDI (RDI‐C/P/D: 24.05 ± 0.64/22.85 ± 0.66/22.61 ± 0.64) than under WLI (WLI‐C/P/D: 22.26 ± 0.60/5.19 ± 0.30/8.62 ± 0.42), regardless of color vision characteristics. This improvement was more pronounced in Type P and Type D and approached Type C in RDI.

**Conclusions:**

Color vision characteristics affect the visibility of blood vessels during ESD, and RDI improves blood vessel visibility regardless of color vision characteristics.

## INTRODUCTION

Color vision deficiency (CVD) is a condition in which different colors are difficult to distinguish. CVD affects as many as 8% of males and 0.4% of females.^1^ The difference in prevalence between males and females reflects the fact that the commonest forms of congenital CVD are inherited in an X‐linked recessive manner.^2^ CVD is divided into protanope, deuteranope, and tritanope, and color appearance differs depending on which cones are abnormal.

In modern society, advances in electronic technologies have led to the widespread use of designs with many colors. In Japan, an awareness campaign initiated by congenital CVD people started around 2000, and efforts have since been made to color designs for universal visibility.^3^ However, there has been scarce evaluation of image visibility in the medical field, particularly for endoscopists with CVD.

Many doctors with CVD are likely unaware of their vision characteristics. In a study investigating the frequency of CVD among 120 medical students, 5.83% were color‐weak, and among the color‐deficient students, 57.0% were protanopic while 43.0% were deuteranopic.^4^ A study examining the prevalence of CVD in medical and dental students compared with non‐medical students found no differences between the two groups.^5^ Additionally, a study from the Netherlands reported that 8% of gastrointestinal endoscopists were diagnosed with CVD.^6^ This study indicates that CVD does not affect the accuracy of endoscopic diagnosis. To our knowledge, this is the only study investigating CVD and endoscopic diagnosis ability. However, given that color is one of the important information in gastrointestinal endoscopy and a certain percentage of endoscopists have CVD from an epidemiological viewpoint, understanding the effect of CVD on endoscopic medicine and devising improvement measures, if needed, will be beneficial for best practice.

Endoscopic submucosal dissection (ESD) is a therapeutic procedure for gastrointestinal tumors and is widely practiced worldwide.^7^ During ESD, it is critical for endoscopists to visually check the blood vessels running through the submucosa before proceeding with the dissection to prevent bleeding.^8^ We found that a trainee preferred a type of image enhancement endoscopy (IEE), namely red dichromatic imaging (RDI), rather than regular white light imaging (WLI) to identify blood vessels during colon ESD. These findings raised clinical questions of whether (1) CVD would affect the visibility of blood vessels during ESD and (2) RDI could improve the visibility of blood vessels during the procedure regardless of color vision characteristics.

IEE is an observation method that converts the wavelength of light in WLI to emphasize the patterns on the mucosal surface of the gastrointestinal tract, the contours of blood vessels, and the color tone.^9^ RDI is one of the new IEE methods that uses three types of light (red, amber, and green) with different characteristics to create deep tissue contrast.^10^ There are three types of RDI modes, each of which produces a characteristic image. Under the basic mode, RDI mode 1, deep blood vessels are depicted in orange for individuals without CVD. In ESD, submucosa injected with indigo carmine appears more vividly blue under RDI than under white light.^11^ Blood vessels in the submucosa are depicted in orange for arteries and red for veins owing to the difference in oxygen saturation. Eventually, the visibility of blood vessels improves, and arteries and veins can be distinguished. Some studies suggest that RDI improves the visibility of blood vessels and bleeding points and shortens the hemostasis time during colon ESD.^12^, ^13^ However, the effect of RDI on endoscopists with and without CVD has not been investigated so far.

Today various computational tools are employed to contribute to progressing universal color designs. One such computer‐based tool is the CVD calibration function within Adobe Illustrator (http://www.adobe.com), which can simulate and display images perceived by people with protanope (Type P) and deuteranope (Type D). Such tools are used in the medical field to simulate visibility for pathologists and ophthalmologists with CVD.^14^, ^15^ Our study aimed to use this computational simulation to investigate how the visibility of submucosal vessels during colorectal ESD procedures varies among people with different color vision characteristics and to examine whether RDI contributes to improving visibility for people with each of the investigated color vision characteristics.

## PATIENTS/MATERIAL AND METHODS

### Study design

The clinical database of Kyorin University Hospital was used for this retrospective study. From April 2021 to March 2023, colorectal ESD was performed on 90 patients with early colorectal cancer, adenomatous lesions, sessile serrated lesions, or neuroendocrine tumors at Kyorin University Hospital. Among these patients, 18 patients had WLI and RDI mode 1 endoscopic images of the same site, which were taken during colorectal ESD (Table 1). ESD of the 18 patients was performed consecutively within the study period, using the EVIS X1 endoscopic system equipped with a PCF‐H290TI endoscope (Olympus, Tokyo, Japan). In these cases, a total of 77 pairs of WLI and RDI endoscopic images were analyzed. The images in each case were anonymized and arranged in the order in which they could have been taken using WLI and RDI in the same scene. The image of the submucosa had at least one blood vessel that the endoscopist should notice. A blood vessel that needs to be identified and distinguished during ESD was selected in each image. Two points on the blood vessel and two points on the background submucosa next to each point on the blood vessel (i.e., two pairs in each image, 154 positions in total) were designated to measure the color difference using Δ*E*_00_. We set each region of interest as a 5‐pixel square on Adobe Photoshop (http://www.adobe.com) to assess Δ*E*_00_. Δ*E*_00_ is a color difference calculated using the color difference formula CIEDE2000,^16^ which was expressed by the CIE (Commission Internationale de l'Eclairage) in 1976 as the distance between two points in the L*a*b* color space and has been improved subsequently. The analysts were Dr. Ohno and Dr. Miyoshi and they were blinded to the clinical demographics of cases when analyzing the data.

**TABLE 1:** Clinical demographics of cases with endoscopic submucosal dissection.

Case	Age	Sex	Location	Lesion size (mm)	Procedure time (min)	Pathology
1	81	Male	C	63	111	Tubular adenocarcinoma
2	51	Male	A	42	50	Tubulovillous adenoma
3	77	Female	T	26	29	Tubulovillous adenoma
4	88	Female	Rb	45	125	Tubular adenocarcinoma
5	63	Male	S	40	90	Tubular adenocarcinoma
6	86	Male	T	25	45	Tubular adenocarcinoma
7	62	Male	C	38	45	Tubular adenocarcinoma
8	54	Female	C	25	35	Sessile serrated lesion
9	72	Male	RS	35	60	Tubular adenocarcinoma
10	63	Male	Ra	53	69	Tubulovillous adenoma
11	84	Male	RS	25	15	Sessile serrated lesion
12	20	Male	Rb	10	22	Neuroendocrine tumor G1
13	65	Female	T	15	20	Sessile serrated lesion
14	55	Male	C	27	40	Tubular adenocaecinoma
15	32	Female	S	50	49	Tubular adenocarcinoma
16	63	Male	Rb	5	17	Neuroendocrine tumor G1
17	56	Female	S	37	60	Tubular adenocarcinoma
18	74	Male	S	15	90	Tubulovillous adenoma

Abbreviations: A, ascending colon; C, cecum; Ra, rectum above the peritoneal reflection; Rb, rectum below the peritoneal reflection; RS, rectosigmoid junction; S, sigmoid colon; T, transverse colon.

### Endoscopic images simulating CVD

**FIGURE 1:**
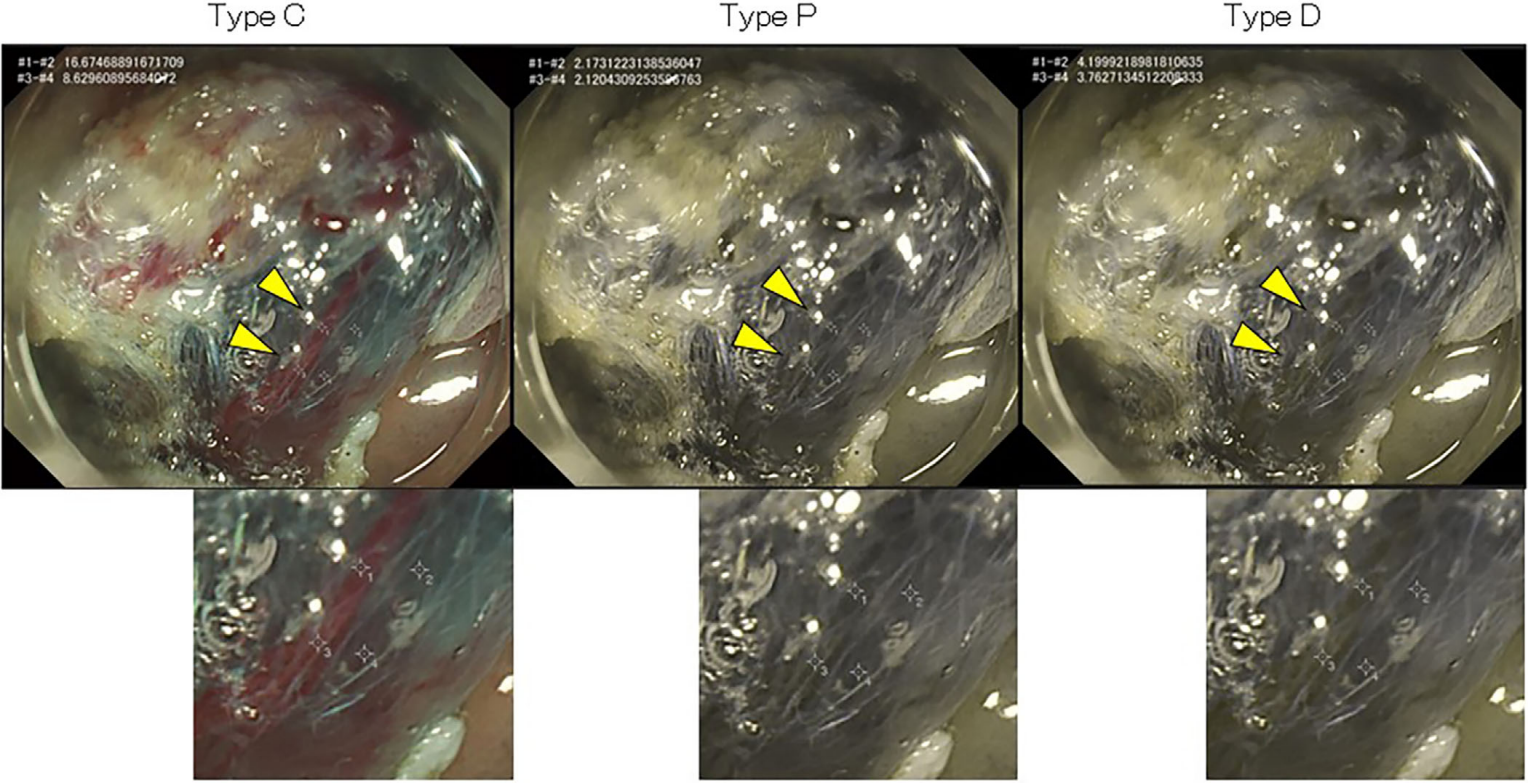
Original white light images obtained during endoscopic submucosal dissection were computationally converted to images simulating vision with color vision deficiency. A representative white light image obtained during endoscopic submucosal dissection and computationally converted images simulating vision with color vision deficiency Types C and D are shown. Blood vessels that appear red in Type C (yellow arrowheads) are depicted darker in Types P and D. Two points on the blood vessel and two points on the background submucosa are shown in the enlarged figure.

**FIGURE 2:**
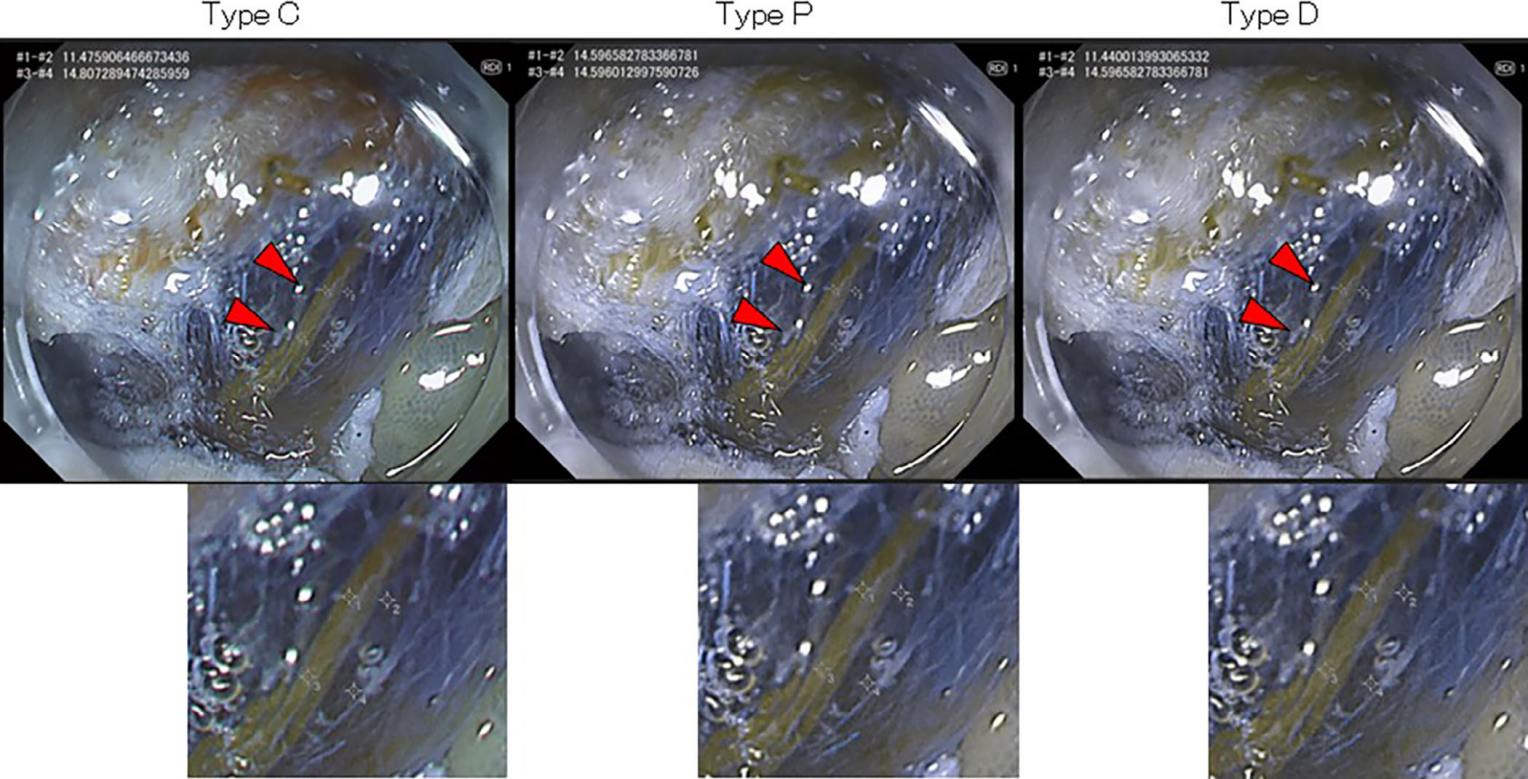
Original images obtained with red dichromatic imaging mode 1 during endoscopic submucosal dissection were computationally converted to images simulating vision with color vision deficiency. A representative image obtained with red dichromatic imaging mode 1 during endoscopic submucosal dissection and computationally converted images simulating vision with color vision deficiency Types C and D are shown. Blood vessels appear yellow in all color vision types (red arrowheads). Two points on the blood vessel and two points on the background submucosa are shown in the enlarged figure.

The recorded WLI and RDI mode1 images were converted to images perceived by people with CVD Type P or D using the color blindness simulation tool built into Adobe Illustrator (Figures 1 and 2). The pre‐conversion images were denoted as WLI‐C and RDI‐C to reflect the observations of people with common color vision, Type C. The post‐conversion images were assigned suffixes P (i.e., WLI‐P and RDI‐P) or D (i.e., WLI‐D and RDI‐D) to reflect the observations of people with CVD Type P or D, respectively.

### Outcomes

The primary endpoint was to assess Δ*E*_00_ between WLI and RDI images in each group of color vision types (C, P, and D). The secondary endpoint was to compare Δ*E*_00_ between WLI and RDI among the groups of color vision types.

### Statistical analysis

Wilcoxon matched‐pairs signed rank test was performed to compare Δ*E*_00_ between WLI and RDI in each group of color vision types (C, P, and D). We used the Friedman test and Dunn's multiple comparisons test to compare color differences between WLI and RDI images among the three groups of color vision types. The criterion of statistical significance was set at *p* < 0.05. IBM SPSS Statistics (ver. 24; IBM) and GraphPad Prism (ver.9.5.1; GraphPad Software) were used for statistical analyses.

## ETHICS STATEMENT

This retrospective study was performed in accordance with the guidelines of the Declaration of Helsinki. This study was approved by the Institutional Review Board of Kyorin University School of Medicine (IRB No. 36) on April 4, 2022. Informed consent was obtained using an opt‐out method.

## RESULTS

### RDI increases the color difference between a blood vessel and the submucosa regardless of color vision characteristics

**FIGURE 3:**
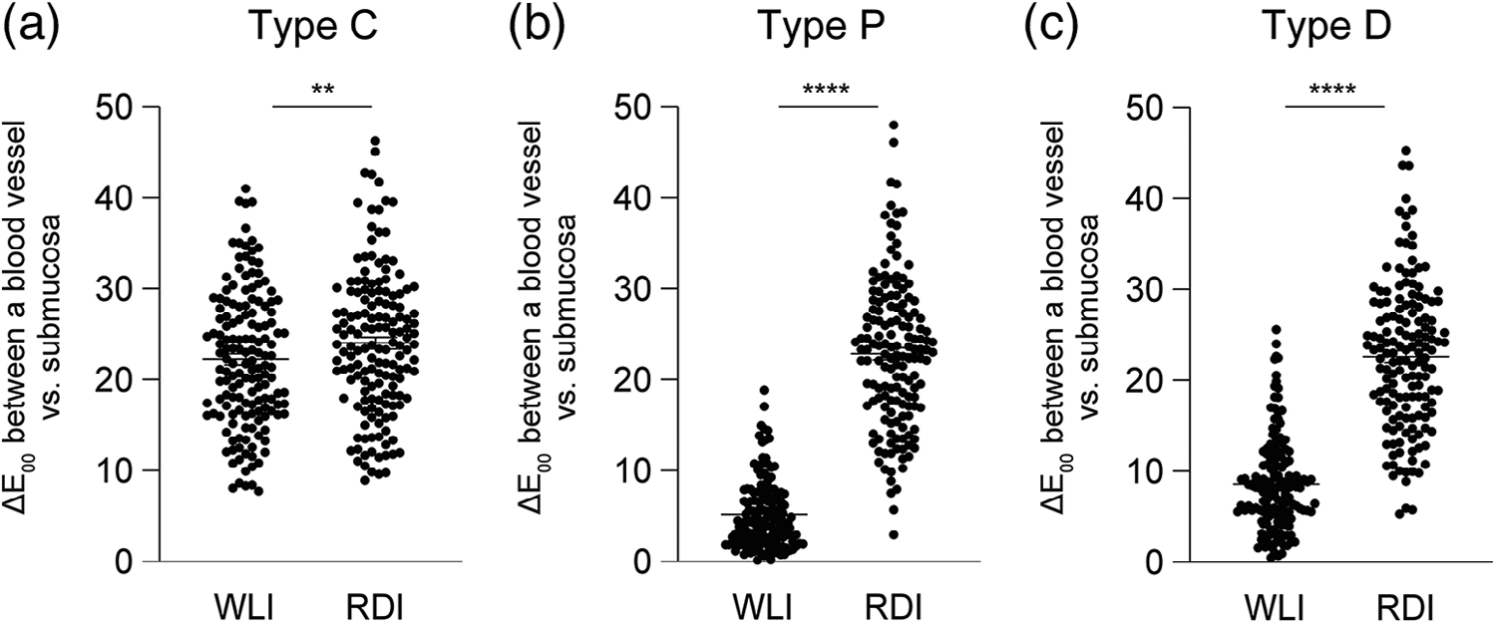
Color difference between a blood vessel and the submucosa in each color vision characteristic. The color differences (Δ*E*_00_) between a blood vessel and the submucosa were measured at two positions in each image of white light imaging (WLI) and red dichromatic imaging. Original images representing Type C (a) and converted images simulating Types P (b) and D (c) were analyzed (*n* = 154 in each group). **p < 0.01 and ****p < 0.0001 with Wilcoxon matched‐pairs signed rank test.

The Δ*E*_00_ between a blood vessel and the background submucosa was evaluated as an indicator of the visibility of the blood vessel. In the Type C group, Δ*E*_00_ was 22.26 ± 0.60 (mean ± SEM) under WLI (WLI‐C) and 24.05 ± 0.64 under RDI (RDI‐C). Therefore, Δ*E*_00_ was significantly larger in RDI‐C images than in WLI‐C images (*p* = 0.0035; Figure 3a). In the Type P group, Δ*E*_00_ was 5.19 ± 0.30 under WLI (WLI‐P) and 22.85 ± 0.66 under RDI (RDI‐P). Therefore, Δ*E*_00_ was significantly larger in RDI‐P images than in WLI images (*p* < 0.0001; Figure 3b). In the Type D group, Δ*E*_00_ was 8.62 ± 0.42 under WLI (WLI‐D) and was 22.61 ± 0.64 under RDI (RDI‐D). Therefore, Δ*E*_00_ was significantly larger in RDI‐D images than in WLI‐D images (*p* < 0.0001; Figure 3c). Overall, these findings indicated that RDI has the potential to improve the visibility of blood vessels regardless of color vision characteristics, and the improvement is more prominent in the CVD groups (Types P and D) than in the Type C group.

### RDI decreased the gap in the color difference between color vision characteristics

**FIGURE 4:**
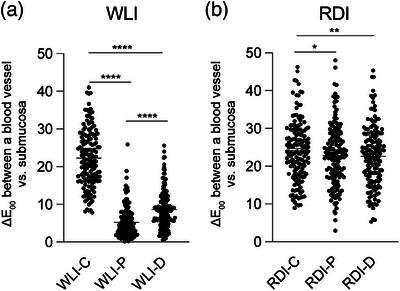
Gaps among color vision characteristics in the color difference between a blood vessel and the submucosa under white light imaging and red dichromatic imaging mode. The color differences (Δ*E*_00_) between a blood vessel and the submucosa under white light image (WLI) (a) and red dichromatic imaging (RDI) (b) were compared among Types C, P, and D. * *p* < 0.05, **p < 0.01, and ****p < 0.0001 with Dunn's multiple comparison test after the Friedman test.

Under WLI, the Δ*E*_00_ of Types C, P, and D were 22.26 ± 0.60, 5.19 ± 0.30, and 8.62 ± 0.42, respectively (Figure 4a). Therefore, the Δ*E*_00_ of Type C was significantly larger than that of Types P and D (*p* < 0.0001 for both comparisons). Moreover, the Δ*E*_00_ of Type D was significantly larger than that of Type P (*p* < 0.0001). Under RDI, the Δ*E*_00_ of Types C, P, and D were 24.05 ± 0.64, 22.85 ± 0.66, and 22.61 ± 0.64, respectively (Figure 4b). Therefore, the Δ*E*_00_ of Type C was significantly larger than those of Types P (*p* = 0.0365) and D (*p* = 0.0091). There was no significant difference in Δ*E*_00_ between Types P and D. All L*a*b* values are shown in Figure S1.

## DISCUSSION

Compared with WLI, RDI improved the Δ*E*_00_ between a vessel and the submucosa for Types C, P, and D and decreased the difference in Δ*E*_00_ among Types C, P, and D. These findings suggest that RDI can contribute to improving the visibility of blood vessels during ESD regardless of color vision characteristics. The results also indicated that RDI during ESD can be beneficial, particularly for endoscopists with Types P and/or D. Improved vascular visibility helps prevent bleeding and contributes to treatment safety. People with Type P CVD see dark red as dark as black. People with Type D CVD have difficulty in seeing green and distinguishing the difference in redness. RDI uses red, amber, and green light with different characteristics to create contrast in deep tissue, with deep blood vessels being represented in orange. Amber is a wavelength of light that is exactly less affected by CVD, so visibility is considered to have improved to the same level as the Type C.

We should note that this is a computational simulation evaluating Δ*E*_00_ as an indicator of the visibility of blood vessels in the submucosa. Our higher brain function is used to recognize objects on the screen during ESD, i.e., we need to understand that color is only one aspect of the information. In this study, through computer simulation, we demonstrated that Δ*E*00 of Types P and D was smaller than Type C. However, this result does not indicate that physicians with Types P and D CVD could experience more difficulty in ESD than those with Type C. Various factors other than blood vessel recognition based on color vision can impact ESD performance and the computer simulation has a limitation in considering these factors. As a future perspective, we plan a study asking individuals with CVD to assess endoscopic images.

Color vision tests are not required for medical licensing in Japan, as they are in the United States and the United Kingdom.^17^ Therefore, given that 80% of Japanese physicians are male^18^ and congenital CVD is more common in males than in females, the prevalence of CVD in Japanese endoscopists may not be low. It is inappropriate to identify an endoscopist's color vision characteristics and link the characteristics to his/her aptitude as an endoscopist. Rather, it is important to recognize that there are endoscopists with various color vision characteristics and to build a system that provides image information to ensure the safety of endoscopic procedures performed by all endoscopists. Furthermore, considering that a certain percentage of medical students and residents have CVD, the concept of universal color design may need to be introduced to learning materials, such as endoscopy atlases and videos. Ron Mace, an advocate of universal design, defines^19^ universal design as “the design of products and environments that can be used by as many people as possible without the need for personal adaptation or special design.” We believe universal design is needed in the endoscopy field.

While this is the first study on the objective assessment of the visibility of blood vessels and the effect of RDI on visibility among people with CVD, there are several limitations. First, we employed computational simulations and Δ*E*_00_ to assess the visibility of blood vessels during ESD. However, the process by which humans recognize objects is extremely complex, and our method does not reflect this complex process. Further studies with the cooperation of subjects with CVD will be needed to address this point. Nevertheless, the present study demonstrated an ESD method for universal visibility, and this can be a pioneer work in the endoscopic field, providing the scientific basis for future clinical studies. Second, we employed the images of ESD cases performed at our single institution. The concentration of indigo carmine in the local injection used for ESD varies among facilities. Some facilities may use a lower concentration of indigo carmine, leading to a lighter blue color of the submucosa, which would affect the result, specifically Δ*E*_00_. However, we use common concentrations (0.005%–0.01%) of indigo carmine in our practice, and thus our results can have general significance. Finally, there are various degrees of CVD, even in each type. Although the computer‐based simulation categorizes major CVD types into Types P and D, each individual with Type P or D could perceive color differently. It is very challenging or even impossible to describe how each individual sees and recognizes colors. This is an unavoidable limitation of studies on color vision.

## CONCLUSION

Our results indicate that the color vision characteristics of individuals affect the visibility of blood vessels during ESD, and RDI improves blood vessel visibility regardless of color vision characteristics. This study provides new insights for introducing the concept of universal color design to the field of endoscopy.

## CONFLICT OF INTEREST DISCLOSURE

Tadakazu Hisamatsu has performed Joint Research with Alfresa Pharma Co. Ltd., EA Pharma Co. Ltd. and received grant support from Mitsubishi Tanabe Pharma Corporation, EA Pharma Co. Ltd., AbbVie GK, JIMRO Co. Ltd., Zeria Pharmaceutical Co. Ltd., Daiichi‐Sankyo, Kyorin Pharmaceutical Co. Ltd., Nippon Kayaku Co. Ltd., Takeda Pharmaceutical Co. Ltd., Pfizer Inc., and Mochida Pharmaceutical Co., Ltd., and consulting and lecture fees from EA Pharma Co. Ltd., AbbVie GK, Janssen Pharmaceutical K.K., Pfizer Inc., Nichi‐Iko Pharmaceutical Co., Ltd., Mitsubishi Tanabe Pharma Corporation, Kyorin Pharmaceutical Co. Ltd., JIMRO Co., Mochida Pharmaceutical Co., Ltd., Gilead Sciences, Kissei Pharmaceutical Co. Ltd., and Takeda Pharmaceutical Co. Ltd.

Jun Miyoshi has received grant support from AbbVie GK, and consulting and lecture fees from EA Pharma Co. Ltd., AbbVie GK, Janssen Pharmaceutical K.K., Jansen Asia Pacific Pte. Ltd., Pfizer Inc., Mitsubishi Tanabe Pharma Corporation, JIMRO Co., Miyarisan Co. Ltd., and Takeda Pharmaceutical Co. Ltd.

Akiko Ohno has received lecture fees and borrowed the equipment necessary for image capture during ESD from Olympus Corporation.

## Supporting information

FIGURE S1 L*a*b* value data of the blood vessel and the background submucosa under white light imaging and red dichromatic imaging mode in each color vision characteristic.
L*a*b values of the blood vessel and the background submucosa that were used to calculate the color differences (ΔE00) are presented. (a) Type C group. (b) Type P group. (c) Type D group.
