# Supplementary figures and images for: The influence of color vision deficiency on vessel visibility during colorectal endoscopic submucosal dissection and the potential advantage of red dichromatic imaging to achieve color vision barrier‐free
Source: DEN Open. 2024 Jul 19;5(1):e410. doi: 10.1002/deo2.410 (PMC11258462; doi:10.1002/deo2.410)

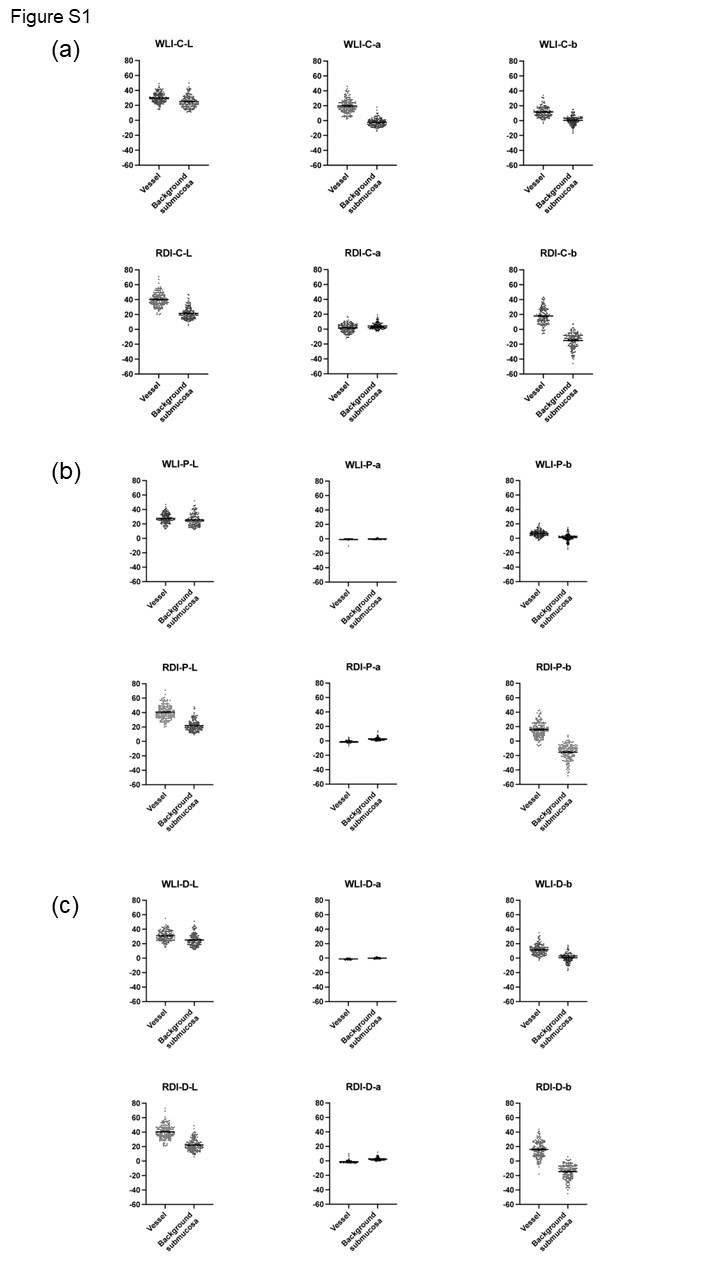

Supplement: Supplementary file 1 — FIGURE S1 L*a*b* value data of the blood vessel and the background submucosa under white light imaging and red dichromatic imaging mode in each color vision characteristic. L*a*b values of the blood vessel and the background submucosa that were used to calculate the color differences (ΔE00) are presented. (a) Type C group. (b) Type P group. (c) Type D group. [file DEO2-5-e410-s001.jpg]
